# The physiology and pathophysiology of cerebrospinal fluid: new evidence

**DOI:** 10.3325/cmj.2021.62.307

**Published:** 2021-08

**Authors:** Marijan Klarica, Milan Radoš, Miroslav Vukić, Darko Orešković

**Affiliations:** 1 Department of Pharmacology and Croatian Institute for Brain Research, Zagreb University School of Medicine, Zagreb, Croatia *marijan.klarica@mef.hr*; 2 Department of Neurosurgery, Clinical Hospital Center Zagreb, Zagreb University School of Medicine, Zagreb, Croatia; 3 Department of Molecular Biology, Ruđer Bošković Institute, Zagreb, Croatia

This issue of the *Croatian Medical Journal* is dedicated to advancements in the diagnostics and treatment of neurosurgical diseases. The issue features some of the articles that were originally envisaged as lectures to be delivered at a joint meeting of Croatian and Japanese Neurosurgical Societies on the topic of cerebrospinal fluid (CSF) physiology and pathophysiology. The meeting entitled Physiology and Pathophysiology of Cerebrospinal Fluid – New Evidence was scheduled to be held in Vodice, Croatia, 2020, but was postponed due to the COVID-19 pandemic. Two parts of the meeting were planned: Cerebrospinal Fluid Volume Regulation and Movement – Revision of Classical Concept and Pathophysiology of Hydrocephalus – New Insights.

The first part was to be dedicated to the role of the choroid plexus in physiology and pathophysiology. The classical concept defines the choroid plexus as the main CSF source (1,2), while a more recent theory, although not negating the importance of the choroid plexus in CSF formation, attributes to this organ a much lesser role than previously believed. The new theory postulates that what is important for CSF formation is the influx and exchange of fluid at the capillary level in the central nervous system (1,3-7). Furthermore, the first part of the meeting aimed to discuss the research on the fate of molecules applied in different parts of the CSF system with or without a blockade of the transport systems in various animal models, from genetically modified mice fetuses (8,9) to large experimental animals (rabbits, cats, dog, pigs) (4-6,10). Mice fetus experiments showed that the movement and fate of molecules in the CSF system were determined by their molecular weight (low-weight molecules move faster), that CSF moved faster in the ventricles than in the subarachnoid space, and that CSF did not circulate (8,9). These observations accord with the new concept of CSF physiology, first published about ten years ago. The new concept draws on research involving big experimental animals showing that CSF was not formed exclusively in the brain ventricles, that it did not unidirectionally move from the ventricles to the subarachnoid space, and that it was not dominantly reabsorbed in the arachnoid granulations of the dural sinuses (4-6). Since substances and metabolites applied in different parts of the CSF system were observed to distribute in all directions, many questions remain regarding drug application in the CNS (2). In addition, advanced radiological techniques provide detailed images of the CNS, with excellent contrast between CSF and the surrounding structures (bones and parenchyma). Therefore, these techniques enable us to precisely segment all CSF spaces and quantify their volumes both in the intracranial and spinal part. Aside from the time spatial inversion pulse (Time-SLIP) method, volumetric MR imaging (for example T2 space and phase-contrast sequences, etc) depicts and even quantifies CSF movement inside the CSF system, especially in regions where this movement is pronounced (the foramen of Monro, mesencephalic aqueduct or cranio-cervical junction) (10,11). Time-SLIP method has shown CSF pulsation but not bulk flow from production site to absorption site (11). The findings of these radiological diagnostic procedures often do not accord with the classical CSF hypothesis. Numerous clinical cases have shown that the choroid plexuses are not necessary for CSF production and that a blocked mesencephalic aqueduct will not always induce hypertensive hydrocephalus, as proposed by the classical concept. Furthermore, contrast applied in the spinal CSF space will almost always be distributed in the direction opposite to the one proposed by the classical concept (10). Finally, a radiological study of arachnoid granulations in patients from birth to 80 years of age strongly suggests that the number, size, and distribution of arachnoid granulations in the superior sagittal sinus and the surrounding cranial bones change significantly over a lifetime (12). Numerous individuals with a completely normal CSF system (without problems in intracranial fluid homeostasis) do not have arachnoid granulations in the dural sinuses. Contrary to what is generally accepted, arachnoid granulations seem not to play an essential role in CSF absorption (12). Thus, the CSF absorption into the venous sinuses and/or lymphatics under physiological conditions, due to their small surface area, should be of minor importance compared with the huge absorptive surface area of the microvessel network of the central nervous tissue (4,5). All the mentioned studies warranted a revision of the classical concept and a development of a new approach to the investigation of CSF physiology.

The second meeting part was planned to deal with different forms of hydrocephalus (acute and subchronic models of aqueductal or cervical blockade or stenosis; knockout models, models of congenital hydrocephalus, kaolin hydrocephalus, idiopathic normal pressure hydrocephalus, arrested hydrocephalus, communicating and noncommunicating hydrocephalus, LAMO, etc). Research in animals (6), newborns (13), and adults (13) showed that acute aqueduct obstruction, with no artifacts of CSF motion through the aqueduct, did not result in hydrocephalus development in a longer time period (6,13). These cases raise the issue of acute hydrocephalus pathophysiology and the accuracy of the classical concept of CSF physiology (14). Transitory acute hydrocephalus in subarachnoid hemorrhage was planned to be discussed as well (11,15). Besides, this part of the symposium would deal with the role of ependymal ciliary motion in the development of congenital hydrocephalus, ie, why this role was earlier perceived to be so important (16). A further important issue is that pressure gradients within the CSF system arising during body posture changes and stenosis placement on different positions do not lead to ventricular enlargement as would be expected according to the classical concept (14,17).

Several lectures were prepared on the pathophysiology of normal pressure hydrocephalus (18-22). Idiopathic normal pressure hydrocephalus (iNPH) is a clinical condition with great variations and without a standard symptom pattern that would clearly separate it from other neurodegenerative diseases. Since CSF drainage treatment yields inconsistent results, the introduction of various CSF biomarkers could improve the clinical approach to this condition (22). The symposium was planned to raise the unresolved questions related to the diagnostics and treatment of hydrocephalus, such as:1) Does every disruption of physiological pulsatile motions of CSF within the CSF system result in ventricular enlargement?; 2) Is a mechanical blockade of the CSF pathway in and of itself enough for hydrocephalus development and why does a clear block of the CSF pathway or a severe stenosis not always lead to a development of acute obstructive hypertensive hydrocephalus?; 3) How often does the CSF movement in the spinal space cause hydrocephalus development?; 4) What is the mechanism of hydrocephalus development in H-Tx rats, DNAH14 knockout mice, and similar experimental models?; 5) What is the pathophysiological significance of increased CSF motion through the aqueduct in the development of iNPH?; 6) What is the predictive role of CSF biomarkers for the development and treatment of different types of hydrocephalus, especially iNPH?; 7) How to differentiate iNPH from the hydrocephalus developing as a result of different neurodegenerative diseases and dementia?; 8) How can modern radiological techniques aid in hydrocephalus research?

The recent understanding of the correlation between CSF physiology and the development of some forms of hydrocephalus should be thoroughly presented, analyzed, evaluated, and discussed. This could bring about new insights into hydrocephalus etiopathology and new treatment approaches that are in accordance with the experimental and clinical data.
